# Expanding the prevalence of Trichophyton indotineae-associated skin infection by transmission from humans to animals

**DOI:** 10.1099/jmm.0.002023

**Published:** 2025-05-23

**Authors:** Ali Abdul Hussein S. AL-Janabi

**Affiliations:** 1 Department of Microbiology, College of Medicine, University of Karbala, Karbala, Iraq

**Keywords:** anthropophilic, *Trichophyton indotineae*, *Trichophyton interdigitale*, *Trichophyton mentagrophytes*

## Abstract

Infection with *Trichophyton indotineae* has been considered an important medical issue in recent times due to the rapid ability of this fungus to develop resistance to different antifungals and its widespread distribution across multiple countries. However, increasing cases of antifungal-resistant infections induce changes in the biological activity of fungi, enabling certain dermatophytes such as *T. indotineae* to infect both animals and humans. This behaviour has certainly had adverse consequences after expanding the host variety of resistant *T. indotineae*. The virulence of *T. indotineae* is predicted to increase, resulting in more difficult treatment for both human and animal infections.

## Introduction

Dermatophytes, common causative agents of skin infection in humans and animals, evolve in a manner similar to other organisms. Changing environmental conditions are the effective factor that allows species with novel characteristics to develop over time. *Trichophyton indotineae* is a new species of dermatophyte that was reclassified from *Trichophyton interdigitale*. It has no morphological or physiological differences from *T. interdigitale* and *Trichophyton mentagrophytes* [1]. The only difference between *T. indotineae* and closely related species is in the internal transcribed spacer (ITS) region of the rDNA, which shows differences in two or three nucleotide positions [2]. Thus, the molecular analysis of the ITS region is commonly used nowadays as an effective tool for the diagnosis of *T. indotineae*.

The primary hosts for *T. indotineae* are humans, as confirmed by hundreds of infections in patients worldwide. *T. indotineae* does not have the tendency to infect animals, and there are only a few registered cases [3,4]. The transfer of *T. indotineae* from humans to animals can pose a serious threat of increased *T. indotineae* infections. This mini-review attempts to discuss this emergency matter.

## Characteristics of *T. indotineae*

*T. indotineae* has recently emerged as a new species with different names, such as *T. mentagrophytes* genotype VIII or *T. mentagrophytes* ITS type VIII. An epidemic in India and a rapid spread to many countries worldwide make it an undeniably serious problem. The close phylogenetic relationship among *T. indotineae*, *T. mentagrophytes*, and *T. interdigitale* complicates the differentiation of their morphological and physiological traits [1]. Such a close relationship has also been proven by the molecular analysis of the high mobility group of domain transcription factor genes [1,5]. All are anthropophilic with remarkable similarities in morphology, physiology and infective nature. Some studies attempted to clarify the differences between these three species based on genotype, phenotype and physiological characteristics. The morphology of all three produces cottony and powdery colonies that expand on culture media as documented by Tang *et al*. [1]. The colours of the colonies ranged between creamy, yellow, yellow-orange and a fluctuating degree of brown. The reverse side of *T. indotineae* colonies was mostly pale brown or yellow-orange, whereas *T. interdigitale* and *T. mentagrophytes* were brown or pale brown. Lipolysis activity is present in 76% of *T. indotineae*, 100% of *T. interdigitale* and 95% of *T. mentagrophytes*. The ability of *T. indotineae* strains to utilize keratin in the hair is lower than that of *T. interdigitale* and *T. mentagrophytes* strains. The urease test for *T. indotineae* and *T. interdigitale* revealed a negative result, compared with a positive result for *T. mentagrophytes* [6,7].

The most efficient method to differentiate *T. indotineae* from other dermatophytes is by conducting a molecular analysis of their genetic contents. ITS sequencing is currently the most common technique used to identify *T. indotineae*. The sequencing of the ITS region revealed that *T. indotineae* differs from *T. mentagrophytes* and *T. interdigitale* in only two or three nucleotide positions [2].

### Humans are the main host of *T. indotineae*

All studies showed that humans are the main host for *T. indotineae*. It is possible that this anthropophilic character is inherited from its parent, *T. interdigitale*. The infection with *T. indotineae* and its recognition as a new species began in India in 2014, before it spread worldwide [1]. Tinea cruris and tinea corporis are common forms of dermatophytoses caused by *T. indotineae* [7]. Recently, infection with *T. indotineae* has become a major issue because its lesion usually tends to spread to various parts of the human body [8]. Resistance to common antifungals, including those used for treating dermatophytoses, is another serious problem with *T. indotineae* infection. The majority of *T. indotineae* strains are resistant to azoles, but they later developed resistance to terbinafine, an allylamine [9]. A survey conducted in five North Indian hospitals between 2014 and 2018 found that 36% of 129 isolates of *T. indotineae* were resistant to not only terbinafine but also fluconazole and griseofulvin [5]. Systemic itraconazole, fluconazole and terbinafine were all ineffective for weeks against a *T. indotineae* isolate that was resistant to multiple drugs [10]. Despite using terbinafine and itraconazole for 2–3 years, two Turkish patients with tinea cruris did not respond to treatment [11]. A man from Switzerland who had *T. indotineae*-induced extensive dermatophytosis did not show improvement after receiving treatment with terbinafine and ketoconazole for 2 months [12]. In Germany, 13 *T*. *mentagrophytes* ITS type VIII strains were found to have resistance to itraconazole and voriconazole [9].

Recently, it was confirmed that terbinafine resistance is a result of a point mutation in the squalene epoxidase (*SQLE*) gene causing a substitution of one or more amino acids in the SQLE protein [13]. Such mutation is a recognized mechanism of resistance to terbinafine in dermatophytes, including *T. indotineae*. The SQLE protein inhibition resulted in a limitation of fungal growth by reducing the amount of ergosterol.

### Transmitted *T. indotineae* from humans to animals

**Fig. 1.**
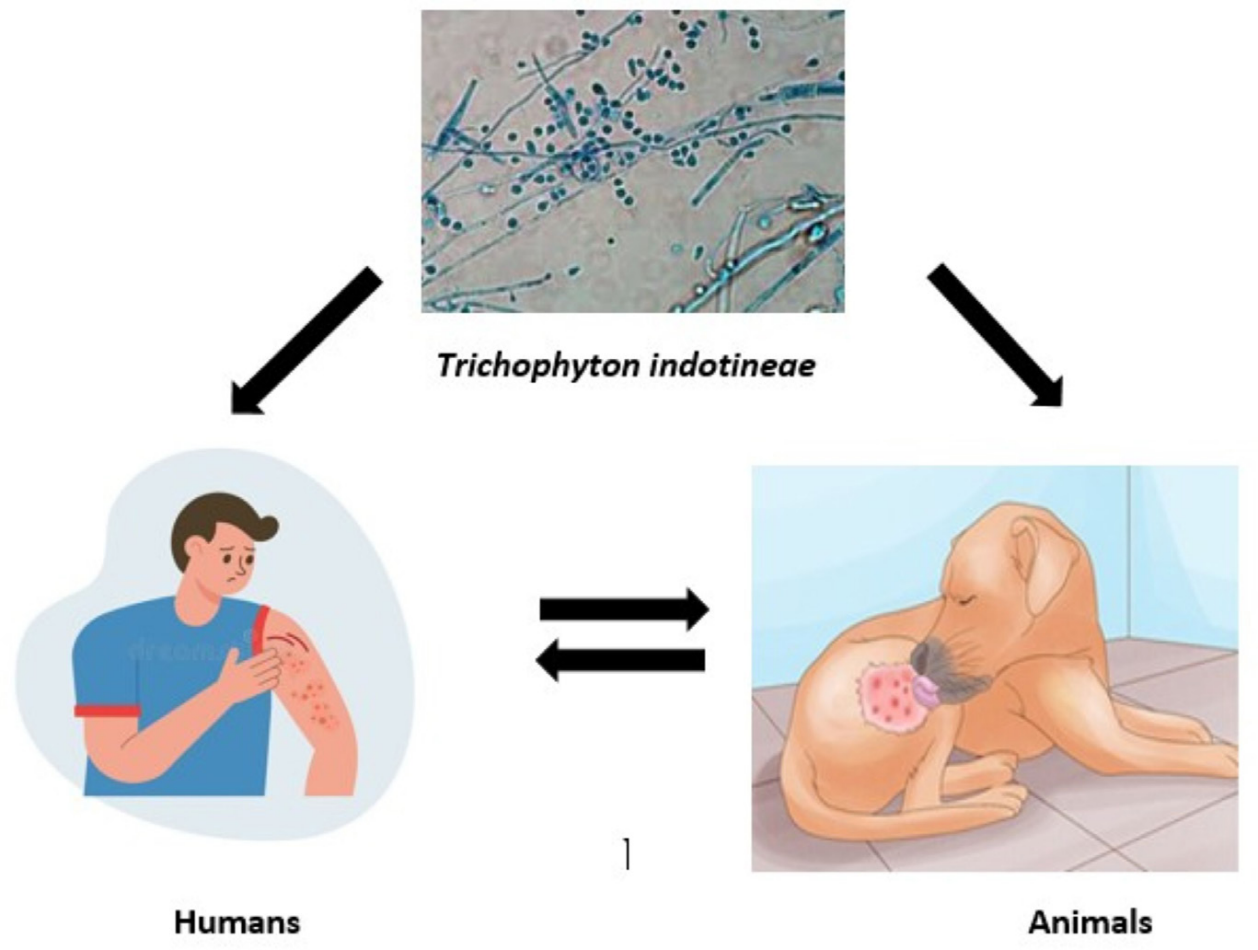
Infection of humans and animals with *T. indotineae* and the possibility of transmission between humans and animals.

The host availability for *T. indotineae* pathogenesis has expanded to include animals other than humans, which is an interesting development (Fig. 1). Thus, it can be predicted that this fungus may become zoophilic in addition to anthropophilic. A dog (2.5 years old) with *T. indotineae* infection was identified in Iran in 2024 [3]. The isolate was resistant to terbinafine (MIC, ≥16 µg ml^−1^) and other antifungals, and had a mutation in the *SQLE* gene that led to the substitution of Phe^397^Leu and Ala^448^Thr. Three wild silver foxes (*Vulpes vulpes*) were diagnosed with terbinafine-resistant *T. mentagrophytes*, which was identified later as *T. indotineae* in Poland [4]. The isolated strains showed high MIC to terbinafine (16 µg ml^−1^) with an amino acid substitution Leu^393^Phe in the SQLE protein. In a study by Jabet et al. [14], an origin of *T. indotineae* infection from animals was found through an analysis of the sequences of ITS regions stored in GenBank and a review of the literature up to March 2021 as part of a study of extensive dermatophytoses caused by *T. indotineae* [14]. An analysis of 526 sequences from 537 showed that 98.8% of them indicated the transmission of *T. indotineae* from humans to humans, while six sequences originated from animals. These six sequences were distributed between two from a survey of 760 calves from Egypt, one from an infected dog from India and three from Poland, but none had any specific animal host indicated.

## Conclusions

Although cases of infected animals with *T. indotineae* are still limited, they can open a new era of dermatophytoses caused by this fungus. The virulence of *T. indotineae*, which causes severe cases of dermatophytoses, coupled with its multidrug resistance ability, cannot be ignored. Extending the host range of *T. indotineae* from humans to animals is a big issue that the world could face in the next generation. It is highly recommended to draw a map of the epidemiology of this fungus in animals, which would be helpful to determine the pattern of distribution and prevent or control the widespread infection among the community. To do this, continuous surveys for *T. indotineae* infections must be conducted among animals, and not just humans.

## Abbreviations

ITS: internal transcribed spacer
SQLE: squalene epoxidase
